# A Systematic Review of the Impact of Antibiotic and Antimicrobial Catheter Locks on Catheter-Related Infections in Adult Patients Receiving Hemodialysis

**DOI:** 10.7759/cureus.45000

**Published:** 2023-09-10

**Authors:** Ayesha Haq, Deepkumar Patel, Sai Dheeraj Gutlapalli, Grethel N Hernandez, Kofi D Seffah, Mustafa Abrar Zaman, Nimra Awais, Travis Satnarine, Areeg Ahmed, Safeera Khan

**Affiliations:** 1 Internal Medicine, California Institute of Behavioral Neurosciences and Psychology, Fairfield, USA; 2 Family Medicine, California Institute of Behavioral Neurosciences and Psychology, Fairfield, USA; 3 Internal Medicine, Richmond University Medical Center, Mount Sinai Health System and Icahn School of Medicine at Mount Sinai, New York City, USA; 4 Internal Medicine/Clinical Research, California Institute of Behavioral Neurosciences and Psychology, Fairfield, USA; 5 Internal Medicine, Piedmont Athens Regional Medical Center, Athens, USA; 6 Internal Medicine, St. George's University School of Medicine, Newcastle Upon Tyne, GBR; 7 Clinical Research, California Institute of Behavioral Neurosciences and Psychology, Fairfield, USA; 8 Pediatrics, California Institute of Behavioral Neurosciences and Psychology, Fairfield, USA; 9 Internal Medicine, California Institute of Neuroscience, Thousand Oaks, USA

**Keywords:** catheter-related bacteremia, anti-locking products, antimicrobial catheter locks, antibiotic catheter locks, hemodialysis, central line-associated infections (clabsi), catheter-related blood stream infection, tunneled dialysis catheter

## Abstract

Central venous catheter (CVC)-based hemodialysis is a major contributor to bacteremia in immunocompromised hosts. Heparin-locking CVCs is a frequent therapeutic procedure. However, it has not been shown to reduce catheter-related bloodstream infections (CRBSIs). For this systematic review, we searched PubMed, PubMed Central, ResearchGate, Science Direct, and Multidisciplinary Digital Publishing Institute (MDPI) for multiple articles published between January 2018 and January 2023 to determine how antimicrobial locking solutions affect CRBSIs, which could ultimately lower the risk of morbidity, mortality, and hospitalization costs. Antilocking products, catheter-related bacteremia, central-line associated bloodstream infections, tunneled dialysis catheter, hemodialysis, antibiotic, and antimicrobial catheter locks, and the Medical Subject Heading (MeSH) method for PubMed were used as the main keywords for searching publications. A pool of 13 studies with 46,139 individuals showed that the therapy group had a lower incidence of CRBSIs than the heparin-treated control group. Furthermore, it was discovered that bacteria were resistant to gentamicin, and the use of antibiotics had no discernible impact on catheter malfunction. In conclusion, the most effective locking solution to date is an antilocking solution made up of an antibiotic or antimicrobial agent combined with low-dose heparin (500-2,500 U/mL).

## Introduction and background

In the United States, kidney illnesses are one of the main causes of death. Approximately 37 million persons in the United States have chronic kidney disease (CKD), and it is estimated that 360 Americans begin dialysis every 24 hours due to renal failure. Arteriovenous fistula (AVF) or central venous catheters (CVCs) are the two methods for performing hemodialysis. Catheter breakage, occlusion, thrombosis, hemorrhage, loss of patency, and catheter-related bloodstream infections (CRBSIs), an infection formed primarily due to CVC insertion rather than seeding microorganisms from another body location, are all negative outcomes of using CVC. Catheter-related bloodstream infections (CRBSIs) have been found to have decreased by 46% in US hospitals from 2008 to 2013 due to advances in medical research. However, it is still thought that 30,100 CRBSIs happen annually in the medical and intensive care units of US acute care hospitals. CRBSIs are a serious cause of morbidity that frequently increase the likelihood of hospitalization, expense, and mortality [1].

There are two types of central lines as follows: (a) tunneled catheters, which are inserted surgically into the internal jugular, subclavian, or femoral vein for long-term (weeks to months) uses like chemotherapy or hemodialysis, and (b) non-tunneled catheters, which are more frequently used. Most CRBSIs are caused by these transcutaneously placed temporary central venous catheters. Within seven to 10 days of CVC implantation, bacteria inhabiting the skin surface begin to build biofilm on the outer surface of the catheter exit site and into the intravascular region. *Staphylococcus aureus*, enterococci, and coagulase-negative staphylococci continue to be the most often identified causal microorganisms. A total of 19-21% of central-line associated bloodstream infections (CLABSIs) reported to the Center for Disease Control and Prevention (CDC) were caused by Gram-negative bacilli [2].

To keep the CVC open, filling the lumen with heparin solutions is normal practice. Heparin reduces the risk of blood coagulation but the formation of biofilms on CVC surfaces is enhanced by heparin [3]. Many anticoagulant intraluminal lock solutions, such as trisodium citrate (TSC), recombinant tissue plasminogen activator (rt-PA), ethylenediaminetetraacetic acid (EDTA), tinzaparin, and urokinase, have been developed during the past 20 years to lessen the complications of CVC. The effects on preventing catheter-related bacteremia are still unknown, even though TSC and EDTA have antibacterial properties through the chelation of iron, calcium, and magnesium [4-6]. Infected catheter removal and reinsertion, systemic antibiotic therapy, and antibiotic or non-antibiotic lock therapy are used to treat CRBSIs. Agents with antibacterial activity, such as antibiotics (cefazolin, gentamicin, cefazolin + gentamicin, minocycline, taurolidine, vancomycin, linezolid, etc.) and ethanol, are now combined with conventional anticoagulant lock solutions to increase the efficacy of lock solutions but the ideal lock composition is still up for dispute. Conflicting findings have been published concerning the emergence of gentamicin resistance. Moreover, no elaborate data regarding the antibiotic effect and bacterial resistance of cefazolin, cefotaxime, vancomycin, minocycline, and linezolid has been found in a pool of our included studies. Similarly, no extensive evidence of the superiority of one antimicrobial agent (EDTA, taurolidine, and citrate) over another was established [7-10].

We conducted a systematic review to evaluate the effectiveness of various lock solutions in reducing CRBSIs in people receiving hemodialysis due to a paucity of research and well-established guidelines on antilock solutions. Our research also aimed to identify the "wonder drug" that can reduce CRBSI-related morbidity and death and determine whether enhanced bacterial resistance is a side effect of antilocking therapy (ALT).

## Review

Methods

The Preferred Reporting Items for Systematic Reviews and Meta-Analyses (PRISMA) 2020 Guidelines have been followed in this systematic review [11].

Literature Search

ResearchGate, ScienceDirect, PubMed, PubMed Central, and Multidisciplinary Digital Publishing Institute (MDPI) databases were used to find studies. The study only considered articles that were published in the English language between January 2018 and January 2023. Keywords used were hemodialysis, CRBSIs, lock solutions, tunneled hemodialysis catheters, bacteremia, and antimicrobial and antibiotic catheter locks. In addition to using these keywords in PubMed, we also used the following strategy to look for pertinent articles in the medical subject heading (MeSH) database of PubMed: "antibiotic prophylaxis/therapeutic use" OR ("central venous catheters/adverse effects"[Majr] OR "central venous catheters/microbiology"[Majr] OR "central venous catheters/pharmacology"[Majr]), additionally, "renal dialysis/instruments" [Majr]. Table 1 shows a summary of our research strategy.

**Table 1:** Search strategy using keywords and MeSH. MDPI: Multidisciplinary Digital Publishing Institute; MeSH: Medical Subject Headings

Search strategy	Database	Number of papers identified	Number of papers after 5-year filter
Antibiotic catheter locks AND hemodialysis	PubMed Central + PubMed	101	17
"Antibiotic prophylaxis/therapeutic use"[Majr] OR ("central venous catheters/adverse effects"[Majr] OR "central venous catheters/microbiology"[Majr] OR "central venous catheters/pharmacology"[Majr] AND "renal dialysis/instrumentation"[Majr]	PubMed	16	2
Antibiotic catheter locks AND hemodialysis	ScienceDirect	215	66
Antibiotic catheter locks AND hemodialysis	ResearchGate	100	26
Antibiotic catheter locks AND hemodialysis	MDPI	2	2

Results

**Figure 1:**
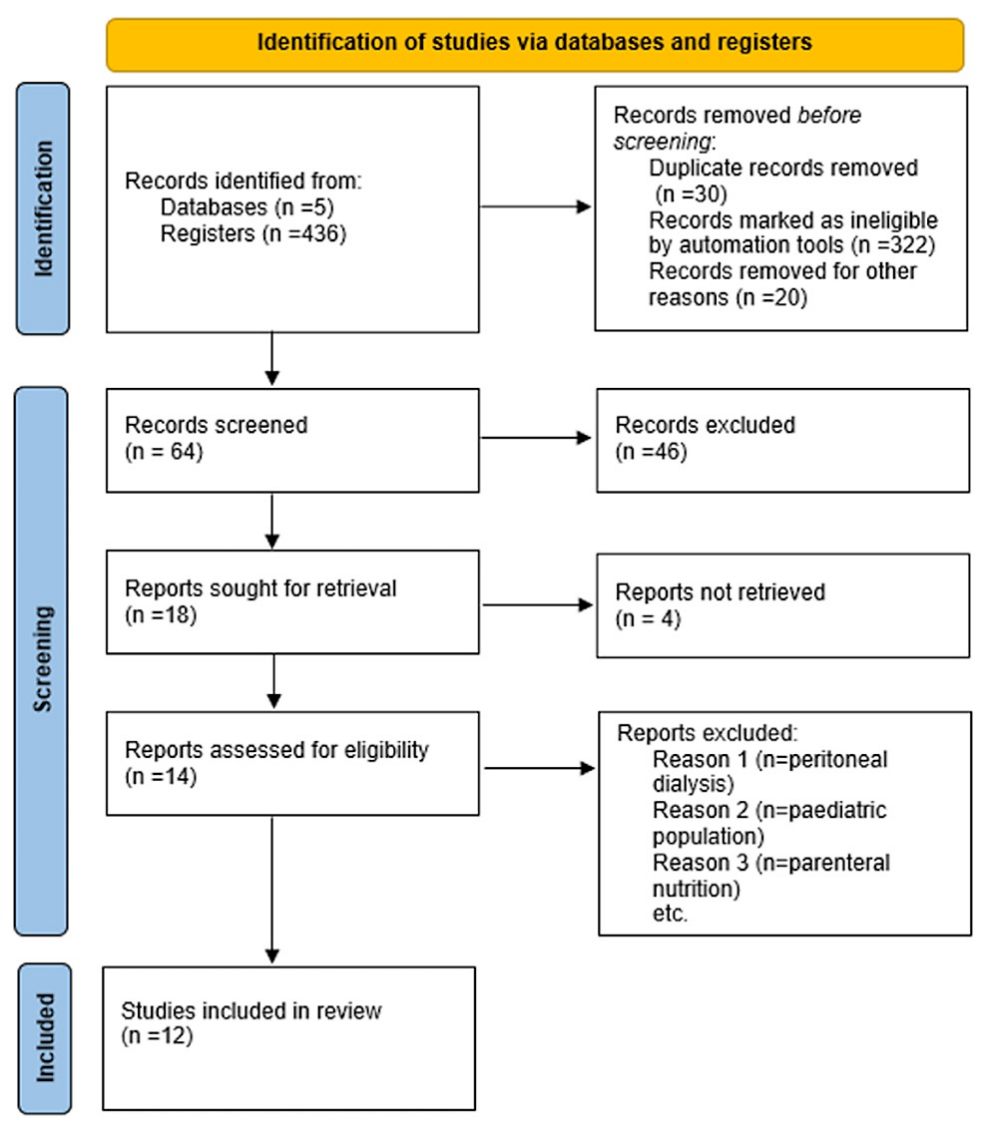
PRISMA flowchart of the included studies for our systematic review. PRISMA: Preferred Reporting Items for Systemic Reviews and Meta-Analysis

A total of 436 documents were located by entering a combination of keywords into the search fields of the aforementioned databases. After applying the automation technique for five years (n=322), eliminating duplicate records (n=30), and other articles (n=20), there were still 64 studies for screening. A total of 46 items were eliminated following the screening procedure, leaving 18 papers available for retrieval. Since four reports could not be retrieved, the number of publications that were eventually left was 14. After quality assessment, the total number of publications that were included in the study was 12 (Figure 1).

Screening of results

The remaining studies underwent a two-phase screening process. During the first stage, papers were included and excluded based on their titles and abstracts. The second stage involved reading full-text papers, followed by applying inclusion criteria. Although reference lists from relevant research were also looked through, no study meeting the PICO (patient/population, intervention, comparison, and outcomes) criteria was found.

Eligibility criteria

Inclusion and Exclusion Criteria

Population: Persons over 18 years of age with end-stage renal disease who received hemodialysis with CVCs were included in the study. Adults having a history of infectious disease, those receiving hemodialysis through an arteriovenous graft, peritoneal dialysis, parenteral nutrition, chemotherapy, pregnant women, and children were excluded from the study.

Intervention: Antibiotic and non-antibiotic central venous lock regimens have been used in this study.

Comparator: Patients receiving standard heparin dose, 4% citrate CVC locks, and systemic antibiotic therapy. CVCs with normal saline solutions were disregarded.

Outcome: Studies had to have a clearly defined primary clinical outcome, such as CRBSIs, and secondary outcomes, such as exit site infections (ESIs) and catheter malfunction, to be considered for this evaluation.

We included publications involving human subjects, peer review, full text, and open access, published within the previous five years (2018-2023) in the English language. Randomized control trials (RCTs), systematic reviews, meta-analyses, narrative reviews, traditional reviews, prospective cohorts, retrospective cohorts, and case reports were eligible studies. Papers using peripheral venous catheters, grey literature, animal subjects, and unpublished articles were disqualified.

Data extraction

To extract the data, Microsoft Excel was employed. The eligible studies were categorized according to the study design, sample size, objectives, and conclusions. Moreover, interventions that were administered, such as antibiotic (AB), antimicrobial agent (non-AB+/- anticoagulant), or a combination of both, were compared to the matched and controlled patients with heparin (HPN), 4% citrate locks, or systematic antibiotic therapy were also a categorizing factor for the included studies. Furthermore, the articles were also studied for CRBSIs, ESIs, and catheter malfunction.

Quality assessment

The nominated studies' quality was evaluated using the appropriate quality rating techniques. Using the Cochrane risk of bias tool described in the Cochrane Handbook, Chapter 8, the risk of bias for RCTs was evaluated (Table 2). Through the assessment of multiple systematic reviews (AMSTAR) checklist, systematic reviews and meta-analyses were evaluated. Case reports, cohort studies, cross-sectional research, and quasi-experimental studies were evaluated using Joanna Briggs Institute (JBI) critical appraisal methods, while narrative and traditional review evaluations were evaluated using the scale for the assessment of non-systematic review articles (SANRA) checklist.

**Table 2:** The risk of bias assessment for RCTs. RCTs: randomized controlled trials

Article	Randomization	Allocation concealment	Blinding (Patients and personnel)	Blinding of outcome (Detection bias)	Incomplete outcome data (Attrition bias)	Selective reporting (Reporting bias)	Other bias
Aniort et al., 2019 [9]	Low risk	Unclear	Moderate risk	Unclear	Low risk	Low risk	Low risk
Winnicki et al., 2018 [12]	Low risk	Low risk	Moderate risk	Low risk	Low risk	Unclear	Low risk

Risk of bias assessment

Studies with low and moderate risk were included after being evaluated using the aforementioned tools. The quality assessment of RCTs is shown in Table 2.

Summary of included studies

This systematic review includes 12 studies that met the eligibility requirements. The included articles are comprehensively summarized in Table 3.

**Table 3:** Summary and characteristics of included studies. AB: antibiotic agent; ALT: antibiotic lock therapy; CRBSI: catheter-related bloodstream infection; CDC: Centers for Disease Control and Prevention; CiT: citrate; EthOH: ethanol; ELT: ethanol lock therapy; EDTA: ethylene diamine tetra acetic acid; ESI: exit site infection; GM: gentamicin; HPN: heparin; KDOQI: Kidney Disease Outcomes Quality Initiative; non-AB: antimicrobial agent; N/A: not applicable; RCTs: randomized controlled trials; rt-PA: recombinant tissue plasminogen activator; TMP: trimethoprim sulfamethoxazole; TRD: taurolidine; TCI: tunneled catheter infection

STUDY	STUDY DESIGN	OBJECTIVE	INTERVENTION ADMINISTERED			CONTROL	CONCLUSION
			AB	Non-AB+/- Anticoagulants	AB+Non-AB		
Bueloni et al. 2019 [7]	Quasi-experimental	Comparison of the effects of taurolidine and citrate against cefazolin and gentamicin in lowering CR-BSI in hemodialysis patients	12 mg/ml cefazolin, 7 mg/ml GM, and 3500 IU/ml HPN	TRD, 4% CiT, and 500 IU/ml HPN	N/A	N/A	Both lock solutions are equally effective at preventing CRBSIs, although patients with antibiotic lock therapy have higher rates of oxacillin-resistant strains.
Mahmood et al. 2020[8]	Descriptive cross-sectional	To observe the frequency of CRBSI and exit site infection in hemodialysis patients with gentamicin lock therapy	N/A	N/A	5,000 IU HPN + 10mg GM in 1ml 0.9% saline in TC 10,000 IU HPN+ 20mg GM in 2ml 0.9% saline in non-TC	N/A	CRBSIs with higher gram-positive bacteria and significant gentamicin resistance were seen often despite the use of ALT.
Aniort et al. 2019[9]	Rrandomized controlled trial	Enoxaparin 1000 U/mL in 40% v/v ethanol's effectiveness and safety in preventing TCI in chronic hemodialysis patients.	N/A	EthOH 40% v/v Enoxaparin 1000 U/ml	N/A	HPN 5000 U/ml or 4% CiT	Ethenox solution is more affordable than other antibacterial substances (taurolidine-citrate). The cost of healthcare could be significantly reduced if TCI is prevented.
Wang et al. 2022[10]	Narrative review	To apply the KDOQI Clinical Practice Guideline for Vascular Access, lock solutions' clinical effectiveness and safety must be evaluated.	G.M. +/- Cefazolin, vancomycin, cefotaxime	4%, 5%, 30%, 46.7% CiT	Vancomycin + HPN/ GM, Cefotaxime + HPN, Minocycline+ EDTA, Cotrimozole+HPN, Taurolock^TM^, Taurolock-Hep 500^TM^, TauroLock^TM^-U25,000, Taurolock^TM^ vs Antibiotic, Ethanol70%/ Heparin, DTA/Ethanol-Ca-EDTA	N/A	Together with rt-PA, 5% citrate (low concentration) can successfully prevent CRBSIs. Antibiotics should be administered at the proper dosage when treating CRBSIs.
Winnicki et al. 2018 [12]	Randomized controlled trial	CRIs, patency, and total expenses of taurolidine-citrate in conjunction with heparin and urokinase against a 4% citrate solution	N/A	TRD	N/A	4% CiT	The 4% citrate-based regimen is inferior to the taurolidine-based regimen in reducing CRBSIs, maintaining catheter patency, and cost-effectiveness.
Salim et al. 2021 [13]	Systematic review and meta-analysis	A review of RCTs examines how antimicrobial and antibiotic lock solutions affect CRBSI incidence.	G.M., cefazolin, vancomycin, cefotaxime, minocycline, TMP	CiT, TRD, EDTA, rt-PA, EtOH	N/A	500, 1000, 5000 U/ml HPN	Only in individuals or facilities with a high risk of CRBSI can antibiotic and antimicrobial lock therapy be employed.
Sheng et al. 2020 [14]		To evaluate the effectiveness and safety of different lock systems.	N/A	Non-AB + CiT/ EDTA/ 5000 U/ml HPN/ Low dose HPN/ Urokinase	N/A	5000 U/ml HPN	For CRBSI prevention, the best lock is an antibacterial agent with low-dose heparin, additional antibiotics, and ethanol.
Chen et al. 2019 [15]		To ascertain the impact of locking solutions on the rate of all-cause mortality in hemodialysis patients, the incidence of CRBSI and ESI, the maintenance of catheter function	N/A	CiT+/-TRD/HPN, EtOH+/- HPN	GM+CiT, TMP/SMX+2500 U/ml HPN	1000, 1666. 2000,2500, 3125, 5000 U/ml HPN	Different locking techniques reduce the occurrence of CRBSI.
Gang et al. 2023[16]	Prospective cohort	To evaluate the effectiveness of ethanol-lock therapy (ELT) in treating CRBSI in the hemodialysis population when combined with systemic antibiotic therapy.	N/A	N/A	Ethanol lock therapy+ Vancomycin+ceftazidime	Vancomycin, ceftazidime	Treatment for CRBSI in H.D. patients includes daily administration of systemic antibiotic medication and short-term ethanol lock therapy.
Hussain et al. 2021 [17]	Retrospective cohort	To evaluate the gentamicin-citrate lock's performance and cost-effectiveness	N/A	N/A	320 mcg GM+4% CiT	HPN	Using G.C. locks lowers hospitalization and CRBSI rates while causing no appreciable increase in gentamicin resistance. G.C. locks are therefore demonstrated to be a cost-effective approach.
Rege et al. 2022 [18]	Case report	To salvage the catheter with the use of Antibiotic lock therapy	Ciprofloxacin	N/A	N/A	N/A	After 14 days of treatment, bacteremia was eradicated with ciprofloxacin antibiotic lock therapy and systemic antibiotic delivery.
Golestaneh et al. 2018 [19]	Traditional review	Examine current clinical practice recommendations for CRBSI in hemodialysis patients.	G.M., tobramycin, minocycline, cefotaxime, vancomycin, cefazolin	4% CiT, TRD+4% CiT, rt-PA, 30%-70% EthOH , EthOH 30%+CiT 4%	N/A	5000 U/ml HPN	It has been demonstrated that following the CDC's clinical practice recommendations helps hemodialysis patients experience fewer CRBSIs and hospitalizations.

Three studies compared lock solutions containing antimicrobial agents (citrate, taurolidine, EDTA, t-PA, and ethanol) with or without anticoagulant agents to antibiotic locks (gentamicin, cefazolin, vancomycin, cefotaxime, minocycline, and trimethoprim-sulfamethoxazole {TMP}) [10,13,19]. Two articles used low to high-dose heparin (500, 1,000, 1,666, 2,0000, 2,500, 3,125, and 5,000 U/mL) as the control locking solution [13,18]. In three of the included papers, locking solutions with antibacterial and anticoagulants were compared to controls consisting of 4% citrate and heparin [9,12,14]. In a study by Chen et al., low to high doses of heparin were employed as a lock therapy compared to locking solutions comprising antibiotics and anticoagulants and antimicrobial and anticoagulant locking solutions [15]. In their review, Wang et al. covered multiple locking systems in various combinations [10].

Catheter-Related Bloodstream and Exit-Site Infections

One of the studies [7] revealed 0.79 and 1.1 CRBSI per 1,000 CVC days in antibiotic and antimicrobial lock solutions, respectively, while six reported 10,417 CRBSIs [8,12-15,17]. A total of 2,448 ESIs were found in three studies [8,14,15], with one research reporting ESI rates of 2.45 and 1.83 per 1,000 CVC days [7]. According to an analysis of these researches, locking solutions with antibiotic and antimicrobial regimens showed a noticeably lower rate of CRBSIs and ESIs than heparin lock treatment alone.

Antibiotic Resistance

On developing gentamicin resistance in locking solutions using gentamicin as the primary agent against bacterial infection, conflicting findings have been published. While Hussein et al. found no gentamicin resistance in their investigation, Mahmood et al. discovered gentamicin resistance in 32 patients [8,17]. Bueloni et al. indicate a higher incidence of oxacillin-resistant bacteria in the antibiotic group [7]. No conclusions could be drawn regarding resistance to antibiotic lock solutions containing vancomycin, cefazolin, minocycline, cefotaxime, and cotrimoxazole. Antimicrobial lock therapy was widely preferred over antibiotic-use lock solutions due to the advantages of a lower risk of bacterial resistance and an equivalent reduction in CRBSIs and ESIs.

Discussion

Bacteria create a dense matrix of polysaccharides that shields them from the immune cells of the immunocompromised host, allowing them to thrive at the catheter insertion site and leading to widespread bacterial infection [13]. According to studies by Bueloni et al., Mahmood et al., Sheng et al., and Hussein et al. S. aureus and coagulase-negative staphylococci are the most common causes of bacteremia in patients receiving hemodialysis, where repeated antibiotic use increases the prevalence of methicillin-resistant strains [7,8,14,17]. According to Gang et al., CRBSIs were only caused by Gram-positive organisms in six patients, while Gram-negative species (Pseudomonas, Burkholderia, Enterobacter, Klebsiella sp., Chromobacterium, Pantoea, Serratia, and Kocuria) caused CRBSIs in 33 patients [16]. According to Mahmood et al., Escherichia coli is the most frequent Gram-negative bacterium to cause CRBSIs [8].

Antibiotic Lock Regimens

According to the recommendations made by the American Society of Diagnostic and Interventional Nephrology, the best locking agent is 1,000 IU/mL heparin [20]. Heparin lock increases while mild doses of citrate (0.2%) prevent biofilm formation on the catheter [19]. Systemic antibiotics do not have strong supporting data for treating or preventing CRBSI [21]. The use of antibiotic and antimicrobial catheter locks as opposed to solo heparin lock therapy for hemodialysis patients is thoroughly examined using data compiled from several research. These regimens may be combined with anticoagulant medications to improve outcomes in reducing CRBSIs, and ESIs, maintaining catheter patency, and preventing bleeding episodes [10,14,15].

The most frequently mentioned antibiotic among the 12 papers considered is gentamicin, an effective locking agent on its own or in conjunction with other antibiotics, antimicrobials, or anticoagulants. Besides gentamicin, the most frequent antibiotics mentioned in the included studies are linezolid, cefazolin, vancomycin, minocycline, and cefotaxime. These antibiotics with antimicrobial and anticoagulant medications have produced some evidence that can serve as the basis for future recommendations for antilocking therapy [7]. According to Sheng et al., the three antilocking medications most frequently used in practice are gentamicin, minocycline, and taurolidine. Their research indicates that the optimum locking solution combines an antibacterial agent with low-dose heparin (500-2,500 U/mL), as this combination stops CRBSIs and bleeding incidents [14]. According to Bueloni et al. and Golestaneh and Mokrzycki, gentamicin is a preventative locking agent against Gram-positive and Gram-negative bacteria [7,19]. Hussein et al. found that 320 μg/mL of gentamicin in 4% citrate resulted in a 70% reduction in CRBSIs [17].

In comparison, Wang et al. reviewed 40 mg/mL of gentamicin in 3.13% citrate as a better locking solution [10]. However, the optimal gentamicin concentration is still up for discussion. According to their analysis, a locking solution containing vancomycin (5 mg/mL) and heparin (2,000 U/mL) reduced CRBSIs by more than 2,000 U/mL of heparin alone. With no impact on ESIs, vancomycin (25 mg/mL) and gentamicin (40 mg/mL) dramatically reduced CRBSI brought on by Gram-positive and Gram-negative microorganisms. Studies using vancomycin as a locking agent showed an 84% reduction in CRBSIs in the antibiotic group compared to patients receiving heparin alone. Another well-known antibiotic mentioned in a collection of our included papers is cefazolin.

Cefazolin was deemed an efficient locking antibiotic in four of 13 studies [7,10,14,19]. For enterococci that are resistant to vancomycin, Wang et al. prefer cefazolin [10]. The combination of gentamicin 5 mg/mL and cefazolin 10 mg/mL significantly decreased the number of CRBSIs in the treatment group. According to Bueloni et al., there was no significant difference in the reduction of CRBSIs between gentamicin + cefazolin and taurolidine-citrate [7]. However, a locking solution including vancomycin and gentamicin outperformed gentamicin + cefazolin. Cefotaxime 10 mg/mL had promising outcomes in reducing CRBSIs in Gram-negative bacteria and patients with nasal S. aureus colonization compared to heparin 5,000 U/mL. Compared to a control group receiving heparin 1,000 U/mL, minocycline 3 mg/mL in combination with EDTA 30 mg/mL or 30% citrate demonstrated protection against CRBSIs caused by S. aureus, Streptococcus, E. coli, and Pseudomonas aeruginosa [21]. Reduced ESIs were reported by Chen et al. in the antibiotic treatment group compared to the control group [15].

Antibiotic Resistance

Antimicrobial drugs are used more frequently in locking therapy because they promise to reduce CRBSIs without fostering bacterial resistance [16]. Gentamicin is the most frequently investigated antibiotic to exhibit resistance bacteria in hemodialysis patients on antilocking medication, according to Salim et al. [13]. They claim that low doses of gentamicin are to blame. At the same time, Sheng et al. attributed bacterial-resistant strains' emergence to repeated antibiotic usage. The evidence is unclear regarding the amount and frequency of administering antibiotics to cause bacterial resistance [14]. According to Bueloni et al., gentamicin at 40 mg/mL and 320 mg/mL did not cause bacterial resistance [7]. In a cohort analysis, neither Hussein et al. nor Fernandez-Gallego et al. found any gentamicin resistance [17,22]. However, Landry et al. found greater morbidity and mortality rates in patients receiving gentamicin (4 mg/mL) + 5,000 IU/mL of heparin therapy for longer than six months due to gentamicin-resistant bacteria [23]. In a cross-sectional study by Mahmood et al., coagulase-negative staphylococci and faecalis were the two most frequent microorganisms to exhibit gentamicin resistance [8]. Even at doses of 1-4 mg/mL, Golestaneh and Mokrzycki reported resistance to gentamicin [19]. The papers included did not mention any clinical evidence of antibiotic resistance associated with cefotaxime, minocycline, or cotrimoxazole [10].

Antimicrobial Lock Regimens

Sheng et al. and Golestaneh and Mokrzycki describe ethanol 30-90% as an efficient antimicrobial locking agent to inhibit biofilm growth on central line catheters without observing any bacterial resistance [14,19]. This is in line with the study of Gang et al., according to their research, ethanol lock therapy and systemic antibiotics, vancomycin, and ceftazidime were more effective at treating CRBSIs than systemic antibiotic treatment alone [16]. According to Wang et al., a catheter lock using 70% ethanol and 5,000 U/mL of heparin once per week was a more effective method for minimizing CRBSIs than using 5,000 U/mL of heparin three times per week [10].

Citrate is a locking solution with antibacterial and anticoagulant capabilities showing greater evidence of controlling bleeding events than heparin [12]. TSC is not in any way superior to heparin 5,000 U/mL in preventing CRBSIs and catheter occlusion. According to Sheng et al. the concentration of TSC solution (1.04-7% and 30%), which should be utilized to reduce CRBSIs, lacks sufficient data. However, in patients who received TSC as a locking agent, bleeding episodes and ESIs were significantly decreased. Another efficient and secure approach uses citrate and other antibiotics, and ethanol [14]. According to Chen et al., citrate-containing regimens significantly reduced CRBSIs and ESIs [15]. A narrative evaluation revealed that citrate lock reduced CRBSIs by 64% and that there was no difference in CRBSIs between 1,500 U/mL of heparin and 46.7% citrate [10]. Citrate should not be used alone; according to Golestaneh and Mokrzycki, CRBSIs and thrombotic events responded well to a combination of 4% citrate, taurolidine, and 500 U/mL of heparin/urokinase 25,000 units [19]. Using taurolidine alone as a locking agent is not recommended because it increases the risk of thrombotic events, which can lead to catheter malfunction. It became a superior locking agent due to its use in conjunction with an anticoagulant (heparin or urokinase). Although TauroLock (taurolidine and 4% citrate) and Neutrolin (taurolidine, heparin, and calcium citrate) are two commercially available locking solutions, Wang et al. do not advise using 4% EDTA alone because it increases the need for thrombolytic medications [10]. Compared to heparin 5,000 U/mL, the administration of trimethoprim, ethanol, and a Ca-EDTA solution significantly reduced the incidence of CRBSIs. There was no discernible therapeutic effect on catheter malfunction, according to four trials [7,10,14,15].

Regarding the influence of the antilocking chemical on catheter malfunction, Gang et al. were unable to draw any conclusions [16]. However, Golestaneh and Mokrzycki claimed that catheter failure was brought on by high concentrations of ethanol (70-100%) [19]. Sheng et al., Hussein et al., and Wang et al. all highlighted increased ototoxicity associated with administering gentamicin without making any clear statements regarding the drug concentration responsible [10,14,17].

Limitations

Despite thoroughly searching for pertinent publications on the research topic, we have not yet been able to add all of them. Our study does not consider mortality in hemodialysis patients, catheter breakage, thrombosis, loss of patency, or the economic effectiveness of alternative locking systems. Furthermore, we could not clearly distinguish one locking agent's superiority over another.

## Conclusions

Our research could be useful in formulating standards for a standard antilocking agent for central line catheters in hemodialysis patients, which is still up for discussion. The most frequent causes of CRBSIs were S. aureus, coagulase-negative staphylococci, and Gram-negative bacteria. The best catheter lock solutions for preventing CRBSIs and ESIs in hemodialysis patients were antibiotics and antimicrobials, especially when combined with anticoagulant medications like low-dose heparin or citrate since they reduced CRBSIs and thrombotic catheter occlusions. Gentamicin, particularly when used with citrate, had the most evidence of reducing CRBSIs among antibiotics. However, gentamicin dose and bacterial resistance to gentamicin are still conflicting matters. There is still a dearth of evidence to support the efficacy of other antibiotics, including vancomycin, minocycline, and cefotaxime, as new medications for this purpose. The most popular anticoagulant with antimicrobial action discussed in our papers' collection is citrate. When coupled with another antibiotic or antimicrobial drug, it outperforms heparin. Antimicrobial medicines like ethanol and taurolidine have demonstrated dramatic reductions in CRBSIs when combined with heparin or urokinase. With the exception of concentrated 70-100% ethanol, no meaningful information was acquired on catheter dysfunction brought on by antibiotic or antimicrobial drugs. In conclusion, CRBSIs are greatly reduced by antibiotic and antimicrobial drugs, but no unique medicine and dosage could be identified.
