# Correction: A Systematic Review of the Impact of Antibiotic and Antimicrobial Catheter Locks on Catheter-Related Infections in Adult Patients Receiving Hemodialysis

**DOI:** 10.7759/cureus.c146

**Published:** 2023-11-21

**Authors:** Ayesha Haq, Deepkumar Patel, Sai Dheeraj Gutlapalli, Grethel N Hernandez, Kofi D Seffah, Mustafa Abrar Zaman, Nimra Awais, Travis Satnarine, Areeg Ahmed, Safeera Khan

**Affiliations:** 1 Internal Medicine, California Institute of Behavioral Neurosciences and Psychology, Fairfield, USA; 2 Family Medicine, California Institute of Behavioral Neurosciences and Psychology, Fairfield, USA; 3 Internal Medicine, Richmond University Medical Center, Mount Sinai Health System and Icahn School of Medicine at Mount Sinai, New York City, USA; 4 Internal Medicine/Clinical Research, California Institute of Behavioral Neurosciences and Psychology, Fairfield, USA; 5 Internal Medicine, Piedmont Athens Regional Medical Center, Athens, USA; 6 Internal Medicine, St. George's University School of Medicine, Newcastle Upon Tyne, GBR; 7 Clinical Research, California Institute of Behavioral Neurosciences and Psychology, Fairfield, USA; 8 Pediatrics, California Institute of Behavioral Neurosciences and Psychology, Fairfield, USA; 9 Internal Medicine, California Institute of Neuroscience, Thousand Oaks, USA

This article has been corrected to remove a protocol that was mistakenly included and referred to as a clinical trial: Ornowska M, Wong H, Ouyang Y, et al.: https://trialsjournal.biomedcentral.com/articles/10.1186/s13063-022-06671-5 Control of Line Complications with KiteLock (CLiCK) in the critical care unit: study protocol for a multi-center, cluster-randomized, double-blinded, crossover trial investigating the effect of a novel locking fluid on central line complications in the critical care population. Trials. 2022, 23: https://trialsjournal.biomedcentral.com/articles/10.1186/s13063-022-06671-5 10.1186/s13063-022-06671-5

The following changes have been made as a result of this removal:

1. The references and citations to the protocol, Ornowska et al., are removed from the tables, the text, and the reference section.
2. All the citation numbers are adjusted accordingly.
3. The PRISMA flow chart shows the updated number of finalized articles included.
4. The quality appraisal table (Table 2) and the summary of characteristics table (Table 3) have been edited accordingly.

The removal of the above protocol did not affect the overall results or the conclusion of the systematic review. The authors regret that this error was not identified prior to submission and publication of this article.

